# Functional Analyses of Cytokinesis Regulators in Bloodstream Stage Trypanosoma brucei Parasites Identify Functions and Regulations Specific to the Life Cycle Stage

**DOI:** 10.1128/mSphere.00199-19

**Published:** 2019-05-01

**Authors:** Xuan Zhang, Tai An, Kieu T. M. Pham, Zhao-Rong Lun, Ziyin Li

**Affiliations:** a Center for Parasitic Organisms, State Key Laboratory of Biocontrol, School of Life Sciences, Sun Yat-sen University, Guangzhou, China; b Department of Microbiology and Molecular Genetics, University of Texas Medical School at Houston, Houston, Texas, USA; University at Buffalo

**Keywords:** bloodstream form, cell division, cytokinesis, *Trypanosoma brucei*

## Abstract

The early divergent protozoan parasite Trypanosoma brucei alternates between the insect vector and the mammalian hosts during its life cycle and proliferates through binary cell fission. The cell cycle control system in T. brucei differs substantially from that in its mammalian hosts and possesses distinct mitosis-cytokinesis checkpoint controls between two life cycle stages, the procyclic form and the bloodstream form. T. brucei undergoes an unusual mode of cytokinesis, which is controlled by a novel signaling cascade consisting of evolutionarily conserved protein kinases and trypanosome-specific regulatory proteins in the procyclic form. However, given the distinct mitosis-cytokinesis checkpoints between the two forms, it is unclear whether the cytokinesis regulatory pathway discovered in the procyclic form also operates in a similar manner in the bloodstream form. Here, we showed that the three regulators of cytokinesis initiation, cytokinesis initiation factor 1 (CIF1), CIF2, and CIF3, are interdependent for subcellular localization but not for protein stability as in the procyclic form. Further, we demonstrated that KLIF, a regulator of cytokinesis completion in the procyclic form, plays limited roles in cytokinesis in the bloodstream form. Finally, we showed that the cleavage furrow-localizing protein FRW1 is required for cytokinesis initiation in the bloodstream form but is nonessential for cytokinesis in the procyclic form. Together, these results identify conserved and life cycle-specific functions of cytokinesis regulators, highlighting the distinction in the regulation of cytokinesis between different life cycle stages of T. brucei.

**IMPORTANCE** The early divergent protozoan parasite Trypanosoma brucei is the causative agent of sleeping sickness in humans and nagana in cattle in sub-Saharan Africa. This parasite has a complex life cycle by alternating between the insect vector and the mammalian hosts and proliferates by binary cell fission. The control of cell division in trypanosomes appears to be distinct from that in its human host and differs substantially between two life cycle stages, the procyclic (insect) form and the bloodstream form. Cytokinesis, the final step of binary cell fission, is regulated by a novel signaling cascade consisting of two evolutionarily conserved protein kinases and a cohort of trypanosome-specific regulators in the procyclic form, but whether this signaling pathway operates in a similar manner in the bloodstream form is unclear. In this report, we performed a functional analysis of multiple cytokinesis regulators and discovered their distinct functions and regulations in the bloodstream form.

## Abstract (precis)

The early divergent protozoan parasite Trypanosoma brucei is the causative agent of sleeping sickness in humans and nagana in cattle in sub-Saharan Africa. This parasite has a complex life cycle by alternating between the insect vector and the mammalian hosts and proliferates by binary cell fission. The control of cell division in trypanosomes appears to be distinct from that in its human host and differs substantially between two life cycle stages, the procyclic (insect) form and the bloodstream form. Cytokinesis, the final step of binary cell fission, is regulated by a novel signaling cascade consisting of two evolutionarily conserved protein kinases and a cohort of trypanosome-specific regulators in the procyclic form, but whether this signaling pathway operates in a similar manner in the bloodstream form is unclear. In this report, we performed a functional analysis of multiple cytokinesis regulators and discovered their distinct functions and regulations in the bloodstream form.

## INTRODUCTION

Cytokinesis is the final step of cell division, which divides the cytoplasm, cellular organelles, and the cell membrane, and is regulated by distinct molecular machineries in different organisms ranging from bacteria to humans. In bacteria and the archaeal species belonging to the Euryarchaeota phylum, cytokinesis requires an FtsZ-based constriction ring structure that is assembled along the short axis of the cell at the cell division site (1). In yeast, amoebae, and animals, an actomyosin ring is assembled at the cell division site, which provides a contractile force that pulls the plasma membrane inward to form an ingressing cleavage furrow (2). In plants, no constriction structure is formed at the cell division site, but a specialized structure termed the phragmoplast, which consists of a bipolar array of microtubules and actin filaments, is assembled at the equator of a dividing cell, where the cell wall is finally built outward to the cell cortex (3). Despite the numerous distinctions in how cytokinesis is regulated among eukaryotic organisms, there is considerable conservation in certain aspects of cytokinesis among plants, yeast, and animals.

The early divergent protozoan Trypanosoma brucei, a member of the Excavata supergroup of eukaryotes and the causative agent of human sleeping sickness, alternates between the insect vector and the mammalian host during its life cycle development. The parasite proliferates through binary cell fission within the hosts and has multiple morphologically distinct life cycle forms, including the procyclic (insect) form and the bloodstream form. T. brucei undergoes an unusual mode of cell division along the longitudinal axis of the cell without the involvement of an actomyosin contractile ring (4). T. brucei is a flagellated unicellular organism, and it assembles a new flagellum during its cell cycle. Both the new and the old flagella are attached to the cell body via a specialized cytoskeletal structure termed the flagellum attachment zone (FAZ) (5). The lengths of the newly assembled flagellum and its associated FAZ appear to determine the cell division plane (6, 7), along which a cell division fold is formed through membrane invagination prior to cleavage furrow ingression (8). Cytokinesis is initiated from the anterior tip of the newly assembled FAZ, and the cleavage furrow ingresses unidirectionally, along the preformed cell division fold, toward the posterior cell end, thereby bisecting the cell into two daughter cells (8, 9). Although the cellular events and the morphological changes during the cell division cycle have been well described (8), the molecular mechanisms underlying many aspects of cytokinesis, such as membrane invagination at the cell division fold, cleavage furrow ingression from the anterior tip of the new FAZ, membrane remodeling at the nascent posterior cell tip, and abscission of the cytoplasmic bridge that connects the two daughter cells, remain unknown and thus require further investigation.

A number of cytokinesis regulatory proteins have been identified and functionally characterized in T. brucei (10–22), most of which are only found in the kinetoplastids that also include Trypanosoma cruzi and Leishmania spp. Only a few of these cytokinesis regulators localize to cytokinesis-associated structures, including the distal tip of the new FAZ, the cleavage furrow, and the cell division fold in the procyclic form (15, 17–23). Through genetic and biochemical analyses, a signaling pathway underlying cytokinesis initiation in the procyclic form has been delineated, which involves two evolutionarily conserved protein kinases, the T. brucei Polo-like kinase (TbPLK) and the T. brucei Aurora B kinase 1 (TbAUK1), and a cohort of trypanosome-specific proteins (18–21). These trypanosome-specific proteins include three regulators of cytokinesis initiation, cytokinesis initiation factor 1 (CIF1), CIF2, and CIF3, which form two separate protein complexes, the CIF1-CIF2 complex and the CIF1-CIF3 complex, and localize to the distal tip of the new FAZ to promote cytokinesis initiation (18–21). These trypanosome-specific proteins also include KLIF and FRW1, which localize to the cleavage furrow (20, 22). KLIF is required for cytokinesis completion in the procyclic form (22), and the function of FRW1 has not been explored.

There is considerable evidence to support the idea that certain aspects of the cell cycle control system differ substantially between the procyclic form and the bloodstream form of T. brucei (24, 25), although the mechanism underlying the distinction remains unclear. The procyclic form appears to lack the mitosis-cytokinesis checkpoint (24, 25), whereas the bloodstream form appears to lack the checkpoint that prevents the cell from entering the next cell cycle before the completion of cytokinesis (8). Moreover, the distinctive positioning of the new flagellum tip between the two life cycle forms (26, 27) may influence cytokinesis initiation in different ways. In the procyclic form, the new flagellum tip is tethered into the old flagellum via a structure termed flagella connector (FC), whereas in the bloodstream form, the FC is absent and the new flagellar tip is embedded within an invagination of plasma membrane in the cell body (26, 27). Given these substantial differences, it is unclear whether the recently delineated cytokinesis signaling pathway operates in a distinct or similar manner in the bloodstream form. In this report, we investigated the function of those cytokinesis regulators in the bloodstream form and uncovered conserved functions for some regulators and life cycle stage-specific functions and regulations for some other regulators. These findings highlight the distinction in the regulation of cytokinesis between different life cycle stages of T. brucei.

## RESULTS

### Cytokinesis initiation factors CIF1, CIF2, and CIF3 are required for cytokinesis initiation in bloodstream trypanosomes.

**FIG 1:**
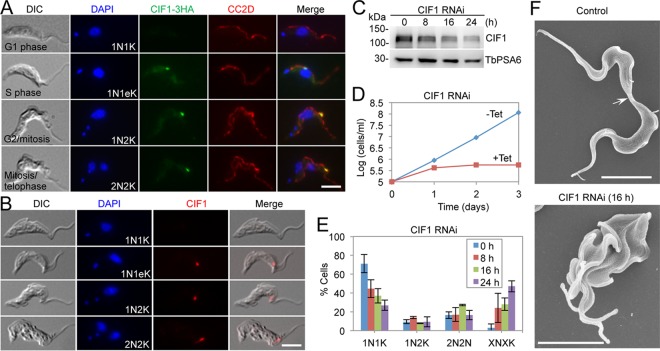
CIF1 is required for cytokinesis initiation in the bloodstream form of T. brucei. (A) CIF1 localizes to the new FAZ tip in bloodstream trypanosomes. CIF1 was endogenously tagged with a triple-HA epitope and was detected by FITC-conjugated anti-HA antibody. The FAZ filament was detected by anti-CC2D antibody. Scale bar = 5 µm. (B) Confirmation of CIF1 localization by immunofluorescence microscopy using anti-CIF1 polyclonal antibody. Scale bar = 5 µm. (C) Western blotting to monitor the efficiency of CIF1 RNAi. CIF1 was detected by anti-CIF1 antibody. TbPSA6 was detected by anti-TbPSA6 antibody and served as the loading control. (D) CIF1 RNAi inhibited cell proliferation in bloodstream trypanosomes. Tet, tetracycline. (E) Knockdown of CIF1 resulted in defective cytokinesis. Shown is the quantitation of cells with different numbers of nuclei (N) and kinetoplasts (K), which were stained with DAPI. A total of 200 cells were counted for each time point, and error bars indicate the standard deviation (SD) of the results from three independent experiments. (F) Scanning electron microscopic analysis of control and CIF1 RNAi cells. The arrow indicates the connecting posterior ends of the two dividing daughter cells among the control cell population. Scale bar = 5 µm.

To determine the subcellular localization of CIF1 in the bloodstream for of T. brucei, we generated a bloodstream-form cell line that expresses CIF1 tagged with a triple-hemagglutinin (3HA) epitope from one of its endogenous loci and then performed immunofluorescence microscopy using anti-HA antibody. CIF1 was undetectable at any subcellular structures in the G_1_ phase, but it was found to localize to the new FAZ tip after the S phase of the cell cycle when the new FAZ was assembled (Fig. 1A), similar to the localization pattern in the procyclic form (18, 20). Immunofluorescence microscopy using anti-CIF1 polyclonal antibody confirmed the results obtained with 3HA-tagged CIF1 (Fig. 1B). To understand the function of CIF1, we carried out RNA interference (RNAi), and Western blotting showed that upon RNAi induction by tetracycline, CIF1 protein level was gradually decreased to ∼30% of the control level at 24 h of RNAi induction (Fig. 1C). Knockdown of CIF1 inhibited cell growth (Fig. 1D), demonstrating that CIF1 is required for cell proliferation in the bloodstream form. Quantitation of cells with different numbers of nuclei (N) and kinetoplasts (K) showed that within 24 h of RNAi induction, cells with multiple nuclei and multiple kinetoplasts (XNXK, X > 2) increased from ∼2% to ∼42% of the total cell population (Fig. 1E), suggesting cytokinesis arrest. Further analysis of these multinucleated cells using scanning electron microscopy (SEM) showed that these cells failed to initiate cytokinesis (Fig. 1F), indicating that CIF1 is required for cytokinesis initiation in the bloodstream form.

**FIG 2:**
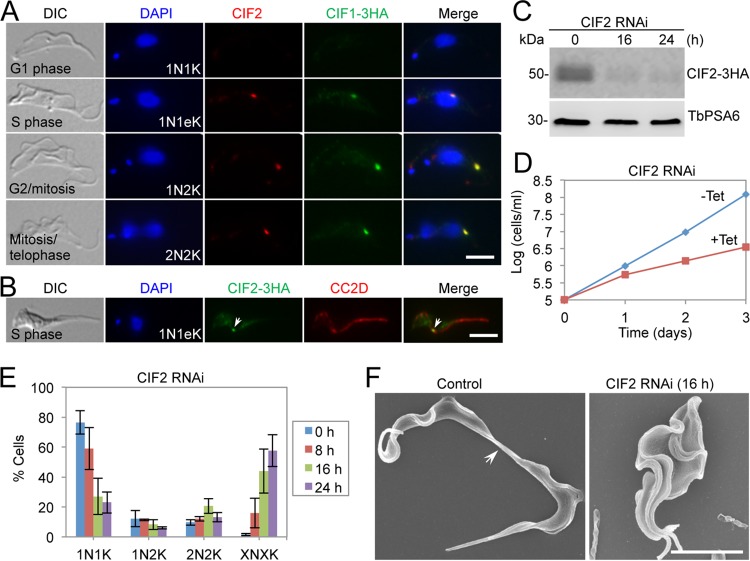
Knockdown of CIF2 in bloodstream trypanosomes caused cytokinesis arrest. (A) CIF2 colocalizes with CIF1 during the cell cycle in bloodstream trypanosomes. CIF2 was detected by anti-CIF2 antibody. CIF1 was endogenously tagged with a triple-HA epitope and was detected by FITC-conjugated anti-HA antibody. Scale bar = 5 µm. (B) CIF2 localizes to the distal tip of the new FAZ. CIF2 was endogenously tagged with a triple-HA epitope and detected by FITC-conjugated anti-HA antibody. FAZ was detected by anti-CC2D polyclonal antibody. The arrow indicates CIF2-3HA signal at the new FAZ tip. Scale bar = 5 µm. (C) Western blotting to monitor the protein level of CIF2 before and after RNAi induction. CIF2 was endogenously tagged with a triple-HA epitope and detected by anti-HA antibody. TbPSA6 served as the loading control. (D) Depletion of CIF2 caused severe growth defects. (E) RNAi of CIF2 caused cytokinesis defects. Shown is the quantitation of cells with different numbers of nuclei (N) and kinetoplasts (K), which were stained with DAPI. A total of 200 cells were counted for each time point, and error bars indicate the SD of the results from three independent experiments (*n *=* *3). X, >2. (F) Scanning electron microscopic analysis of control and CIF2 RNAi cells. The arrow indicates the connecting posterior ends of the two dividing daughter cells among the control cell population. Scale bar = 5 µm.

To examine the subcellular localization of CIF2 and its potential colocalization with CIF1, we generated an anti-CIF2 polyclonal antibody in rabbit and performed coimmunofluorescence microscopy using the cells expressing CIF1-3HA. CIF2 was not detectable in G_1_ cells, but it colocalized with CIF1 from the S phase onward (Fig. 2A). Coimmunofluorescence of cells expressing CIF2-3HA with anti-HA antibody and anti-CC2D antibody, which labels the FAZ, showed that CIF2 localizes to the new FAZ tip (Fig. 2B). Inducible RNAi was then performed to study the function of CIF2 in the bloodstream form, and Western blotting showed that after RNAi induction for 16 h, the level of CIF2, which was endogenously tagged with a triple-HA epitope, was reduced to ∼10% of the control level (Fig. 2C). This depletion of CIF2 caused severe growth defects (Fig. 2D) and led to an accumulation of cells containing multiple (>2) nuclei and multiple (>2) kinetoplasts (XNXK, X > 2) at ∼60% of the total cell population after RNAi induction for 24 h (Fig. 2E), indicating defective cytokinesis. Scanning electron microscopy (SEM) analysis of these multinucleated cells showed that they failed to initiate cytokinesis (Fig. 2F). These results demonstrated that CIF2 is required for cytokinesis initiation in the bloodstream form.

**FIG 3:**
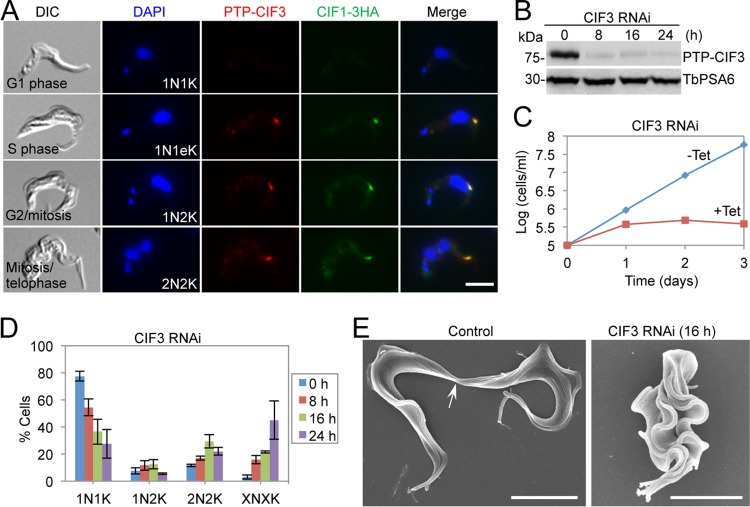
Depletion of CIF3 by RNAi arrests cytokinesis in bloodstream trypanosomes. (A) CIF3 colocalizes with CIF1 during the cell cycle in the bloodstream form. Cells coexpressing endogenously PTP-tagged CIF3 and 3HA-tagged CIF1 were coimmunostained with anti-protein A antibody and anti-HA antibody. Scale bar = 5 µm. (B) Western blotting to monitor the efficiency of CIF3 RNAi. CIF3 was endogenously tagged with an N-terminal PTP epitope and was detected by anti-protein A antibody. TbPSA6 served as the loading control. (C) Depletion of CIF3 caused growth inhibition. (D) Depletion of CIF3 resulted in cytokinesis defects. Shown is the quantitation of cells with different numbers of nuclei (N) and kinetoplasts (K), which were stained with DAPI. A total of 200 cells were counted for each time point, and error bars indicate the SD of the results from three replicates (*n *=* *3). X, >2. (E) Scanning electron microscopic analysis of control and CIF3 RNAi cells. The arrow indicates the connecting posterior ends of the two dividing daughter cells among the control cell population. Scale bar = 5 µm.

We next examined the subcellular localization of CIF3 and its potential colocalization with CIF1. To this end, we generated a cell line coexpressing the PTP-tagged CIF3 and 3HA-tagged CIF1 from their respective endogenous loci. Coimmunostaining with anti-protein A antibody, which detects PTP-CIF3, and anti-HA antibody, which detects CIF1-3HA, showed that CIF3 was not detectable in G_1_ cells, but it colocalized with CIF1 from the S phase onward (Fig. 3A). RNAi of CIF3 resulted in a rapid decrease in CIF3 protein to ∼5% of the control level after 8 h of RNAi induction (Fig. 3B) and inhibited cell growth (Fig. 3C), suggesting that CIF3 is essential for cell proliferation. Quantitation of cells with different numbers of nuclei and kinetoplasts showed that the depletion of CIF3 led to an increase in cells with multiple (>2) nuclei and multiple (>2) kinetoplasts (XNXK, X > 2) to ∼40% of the total population after 24 h (Fig. 3D), indicating that cytokinesis was inhibited. Further analysis of these multinucleated cells using SEM showed that these cells failed to initiate cytokinesis (Fig. 3E). Together, these results demonstrated that CIF3 is required for cytokinesis initiation in the bloodstream form.

### FRW1 is required for cytokinesis initiation in the bloodstream form but not the procyclic form.

**FIG 4:**
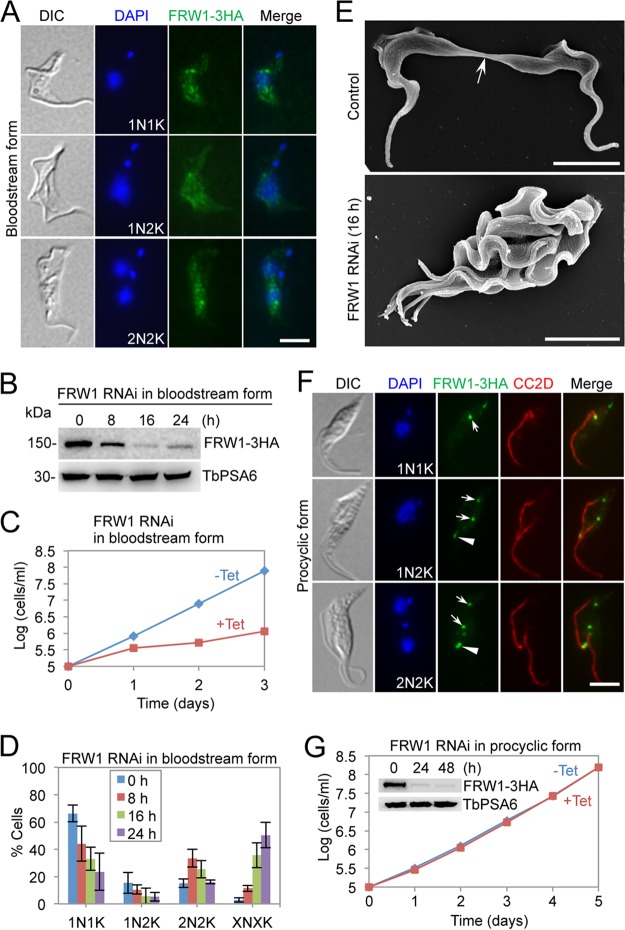
Knockdown of FRW1 in bloodstream trypanosomes caused cytokinesis arrest. (A) Subcellular localization of FRW1 in the bloodstream form. FRW1 was endogenously tagged with a triple-HA epitope and detected by FITC-conjugated anti-HA antibody. Scale bar = 5 µm. (B) Western blotting to monitor the protein level of FRW1 before and after RNAi induction. FRW1, endogenously tagged with a triple-HA epitope, was detected by anti-HA antibody. TbPSA6 served as the loading control. (C) Depletion of FRW1 caused growth inhibition in bloodstream trypanosomes. (D) RNAi of FRW1 arrested cytokinesis. Shown is the counting of cells with different numbers of nuclei (N) and kinetoplasts (K), which were stained with DAPI. A total of 200 cells were counted for each time point, and error bars indicate the SD of the results from three independent experiments (*n *=* *3). X, >2. (E) Scanning electron microscopic analysis of control and FRW1 RNAi cells. The arrow indicates the connecting posterior ends of the two dividing daughter cells among the control cell population. Scale bar = 5 µm. (F) Subcellular localization of FRW1 in the procyclic form. Endogenously 3HA-tagged FRW1 was detected by FITC-conjugated anti-HA monoclonal antibody (MAb). Scale bar = 5 µm. Arrows indicate FRW1 fluorescence signal at the flagellar pocket region, whereas arrowheads indicate the FRW1 signal at the new FAZ tip. Scale bar = 5 µm. (G) Ablation of FRW1 by RNAi in the procyclic form exerted no effect on cell proliferation. The inset shows the Western blotting of 3HA-tagged FRW1 before and after RNAi induction. TbPSA6 served as the loading control.

FRW1 is a CIF1-interacting protein, and its function has not been characterized (20). We examined the subcellular localization of FRW1 in the bloodstream form by immunofluorescence microscopy using cells expressing endogenously 3HA-tagged FRW1. The results showed that FRW1 was detected as multiple punctate dots in the middle portion of the cells (Fig. 4A). RNAi of FRW1 in the bloodstream form resulted in a decrease in the level of FRW1 to ∼10% of the control level at 16 h, but its level slightly recovered at 24 h of RNAi induction (Fig. 4B). The depletion of FRW1 caused several growth defects (Fig. 4C), indicating that FRW1 is essential for cell proliferation in the bloodstream form. Quantitation of cells with different numbers of nuclei and kinetoplasts showed that FRW1 knockdown caused an increase in multinucleated cells (XNXK, X > 2) to ∼50% of the total cell population after 24 h of RNAi (Fig. 4D), suggesting defective cytokinesis. SEM analysis of these multinucleated cells showed that they had failed to initiate cytokinesis (Fig. 4E). These results demonstrated that FRW1 is required for cytokinesis initiation in the bloodstream form. In contrast, in the procyclic form, FRW1 localizes to the flagellar pocket region in G_1_ cells and is concentrated at the new FAZ tip from S phase to telophase (Fig. 4F) and at the cleavage furrow during cytokinesis (20). RNAi of FRW1 in the procyclic form depleted FRW1 protein in 24 h of RNAi (Fig. 4G, inset), but the depletion of FRW1 did not affect cell proliferation (Fig. 4G), indicating that FRW1 is not essential for cell division in the procyclic form. These results suggest functional distinctions of FRW1 between the two life cycle forms.

### Knockdown of KLIF in bloodstream trypanosomes does not cause any detectable cytokinesis defects.

**FIG 5:**
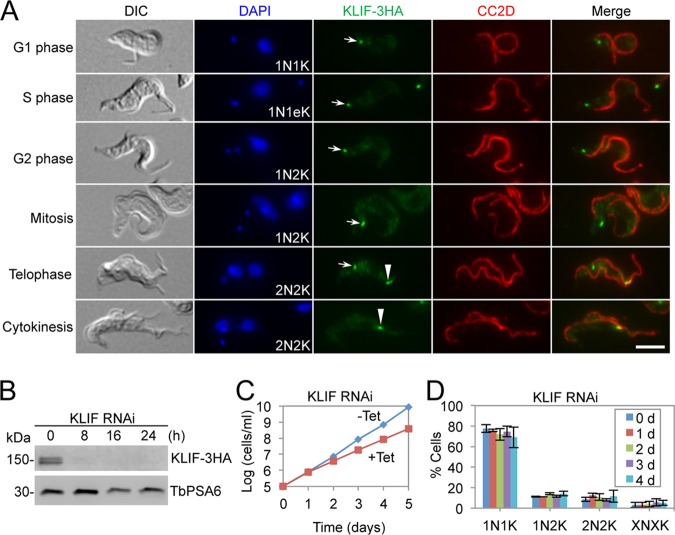
KLIF is not required for cytokinesis in bloodstream trypanosomes. (A) Subcellular localization of KLIF in bloodstream trypanosomes. KLIF was endogenously tagged with a triple-HA epitope and detected by FITC-conjugated anti-HA antibody. The FAZ filament was detected by anti-CC2D antibody. Arrows indicate the KLIF signal at the flagellar pocket region, whereas arrowheads indicate the KLIF signal at the new FAZ tip (telophase) and the cleavage furrow (cytokinesis). Scale bar = 5 µm. (B) Western blotting to monitor the efficiency of KLIF RNAi. KLIF was endogenously tagged with a triple-HA epitope and was detected by anti-HA antibody. TbPSA6 served as the loading control. (C) Depletion of KLIF caused a moderate growth defect. (D) Depletion of KLIF exerted no specific effect on cell cycle progression in bloodstream trypanosomes. Shown is the quantitation of cells with different numbers of nuclei (N) and kinetoplasts (K), which were stained with DAPI. A total of 200 cells were counted for each time point, and error bars indicate the SD of the results from three replicates (*n *=* *3). X, >2.

KLIF is a novel kinesin-tropomyosin combination protein localizing to the new FAZ tip and the cleavage furrow in the procyclic form of T. brucei (20, 22). Knockdown of KLIF in the procyclic form caused a moderate defect in cytokinesis completion (22). We investigated the localization and function of KLIF in the bloodstream form. Immunofluorescence microscopy showed that 3HA-tagged KLIF was detected as a dot near the flagellar pocket in cells from G_1_ phase to telophase and was detected at the new FAZ tip at telophase and at the cleavage furrow during cytokinesis (Fig. 5A). This localization pattern is similar to that in the procyclic form (20). RNAi of KLIF resulted in a decrease in KLIF protein to undetectable levels from 8 h of RNAi induction (Fig. 5B) and caused a moderate growth defect (Fig. 5C), similar to the growth defect in the KLIF-depleted procyclic form (22). However, quantitation of cells with different numbers of nuclei and kinetoplasts showed that there was no specific defect in cell cycle progression (Fig. 5D). These results suggest that KLIF is not required for cytokinesis in the bloodstream form.

### Investigation of the potential functional interdependence among CIF1, CIF2, and CIF3.

**FIG 6:**
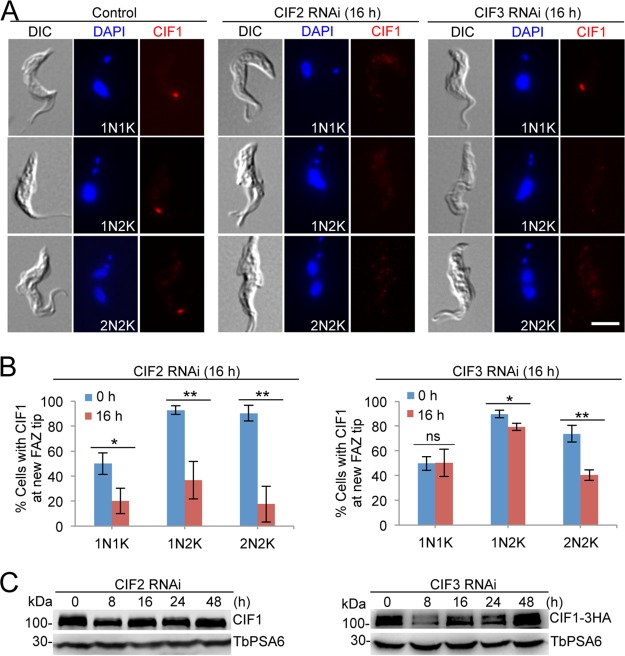
Localization of CIF1 to the new FAZ tip requires CIF2 and CIF3. (A) Subcellular localization of CIF1 in control cells, CIF2 RNAi cells, and CIF3 RNAi cells. CIF1 was detected by anti-CIF1 antibody. RNAi was induced for 16 h. Scale bar = 5 µm. (B) Quantitation of cells with CIF1 fluorescence signal at the new FAZ tip among cells with different numbers of nuclei (N) and kinetoplasts (K), which were stained with DAPI. A total of 200 cells were counted for each time point, and error bars indicate the SD of the results from three independent experiments (*n *=* *3). *, *P < *0.05; **, *P < *0.01; ns, no statistical significance. (C) Western blotting to monitor the protein level of CIF1 in control cells, CIF2 RNAi cells, and CIF3 RNAi cells. In CIF2 RNAi cell line, CIF1 was detected by anti-CIF1 antibody. In CIF3 RNAi cell line, CIF1 was endogenously tagged with a triple-HA epitope and detected by anti-HA antibody. TbPSA6 served as the loading control.

Given the functional interdependence among CIF1, CIF2, and CIF3 in the procyclic form (19, 21, 28), we sought to investigate their functional relationships in the bloodstream form. We first examined the effect of CIF2 knockdown and CIF3 knockdown on the subcellular localization and stability of CIF1 by immunofluorescence microscopy and Western blotting. Knockdown of CIF2 disrupted CIF1 localization to the new FAZ tip in all cell types (Fig. 6A and B). Knockdown of CIF3, however, disrupted the localization of CIF1 to the new FAZ tip during the G_2_ and mitotic phases (1N2K and 2N2K cells, respectively) but not at S phase (1N1K cells) (Fig. 6A and B). Despite the strong effect on CIF1 localization, the CIF1 protein level appeared to be largely unaffected by the depletion of CIF2 (Fig. 6C). The depletion of CIF3, however, caused a light decrease in the CIF1 protein level at 8 h of RNAi, but the level of CIF1 protein gradually restored after 16 h of RNAi (Fig. 6C). These results suggest that CIF2 is required for targeting CIF1 to the new FAZ tip from the S phase of the cell cycle, whereas CIF3 functions to maintain CIF1 at the new FAZ tip from the G_2_ phase of the cell cycle.

**FIG 7:**
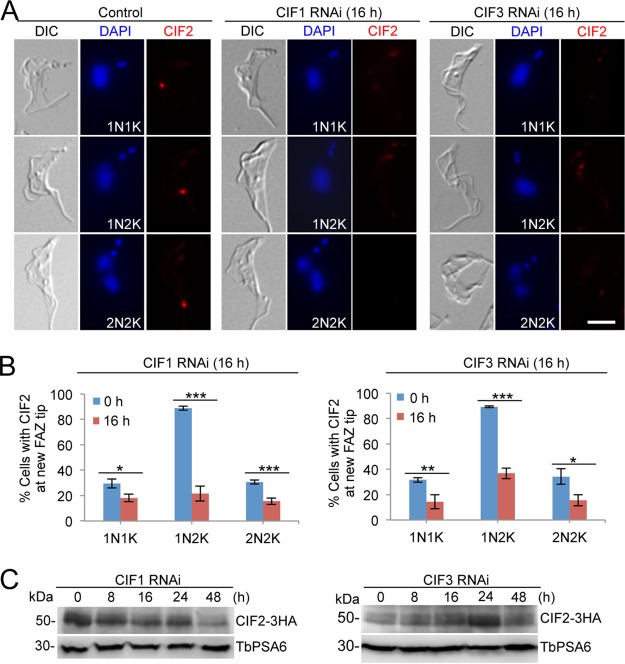
Localization of CIF2 to the new FAZ tip requires CIF1 and CIF3. (A) Subcellular localization of CIF2 in control cells, CIF1 RNAi cells, and CIF3 RNAi cells. CIF2 was detected by anti-CIF2 antibody. RNAi was induced for 16 h. Scale bar = 5 µm. (B) Quantitation of cells with CIF2 fluorescence signal at the new FAZ tip among cells with different numbers of nuclei (N) and kinetoplasts (K). A total of 200 cells were counted for each time point, and error bars indicate the SD of the results from three independent experiments (*n *=* *3). *, *P < *0.05; **, *P < *0.01; ***, *P* < 0.001. (C) Western blotting to monitor the protein level of CIF2 in control cells, CIF1 RNAi cells, and CIF3 RNAi cells. CIF2 was endogenously tagged with a triple-HA epitope and detected by anti-HA antibody. TbPSA6 served as the loading control.

The effect of depletion of CIF1 and CIF3 on the subcellular localization and stability of CIF2 was similarly investigated by immunofluorescence microscopy and Western blotting. Knockdown of CIF1 and CIF3 impaired the localization of CIF2 to the new FAZ tip in all cell types (Fig. 7A and B). Western blotting showed that knockdown of CIF1 caused a slight decrease in CIF2 protein level from 16 h of CIF1 RNAi and further decreased the CIF2 level to ∼20% of the control level at 48 h (Fig. 7C). However, knockdown of CIF3 did not affect CIF2 protein level (Fig. 7C). Together, these results suggest that CIF1 and CIF3 are both required for CIF2 localization to the new FAZ tip from the S phase of the cell cycle. However, given the reduction in CIF2 protein level after CIF1 RNAi (Fig. 7C), CIF1 may play a role in maintaining CIF2 stability.

**FIG 8:**
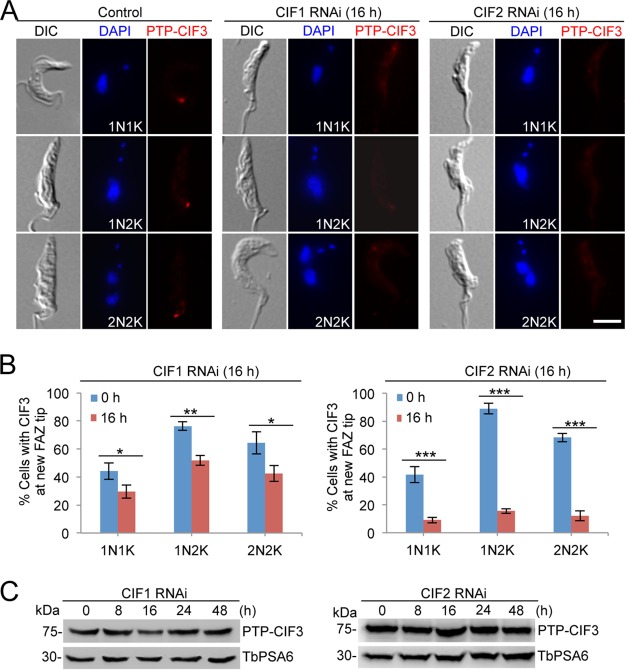
Localization of CIF3 to the new FAZ tip depends on CIF1 and CIF2. (A) Subcellular localization of CIF3 in control cells, CIF1 RNAi cells, and CIF2 RNAi cells. CIF3 was endogenously tagged with an N-terminal PTP epitope and detected by anti-protein A antibody. RNAi was induced for 16 h. Scale bar = 5 µm. (B) Quantitation of cells with CIF3 fluorescence signal at the new FAZ tip among cells with different numbers of nuclei (N) and kinetoplasts (K). A total of 200 cells were counted for each time point, and error bars indicate the SD of the results from three independent experiments (*n *=* *3). *, *P < *0.05; **, *P < *0.01; ***, *P* < 0.001. (C) Western blotting to monitor the protein level of CIF3 in control cells, CIF1 RNAi cells, and CIF2 RNAi cells. PTP-CIF3 was detected by anti-protein A antibody. TbPSA6 served as the loading control.

Finally, we investigated the effect of CIF1 RNAi and CIF2 RNAi on the localization and stability of CIF3 by immunofluorescence microscopy and Western blotting. To this end, CIF3 was endogenously tagged with an N-terminal PTP epitope in cells harboring the CIF1 RNAi construct or the CIF2 RNAi construct. Knockdown of CIF1 or CIF2 disrupted the localization of CIF3 to the new FAZ tip in all cell types (Fig. 8A and B), and Western blotting showed that the level of PTP-tagged CIF3 protein was not significantly changed in CIF1 RNAi cells and CIF2 RNAi cells (Fig. 8C). These results suggest that localization of CIF3 to the new FAZ tip requires both CIF1 and CIF2.

## DISCUSSION

The cell cycle control system and its regulatory machineries in T. brucei possess a number of unusual features (4, 29), and there appears to be substantial differences in cell cycle control between different life cycle stages, such as the procyclic form and the bloodstream form (8, 24, 25, 30). In the procyclic form, ablation of mitotic regulators inhibited mitosis but not cytokinesis, mirroring the effects caused by the antitubulin drug rhizoxin (31), whereas in the bloodstream form, ablation of mitotic regulators inhibited both mitosis and cytokinesis but allowed cells to enter the next rounds of DNA replication and organelle duplication (24, 25, 30). These findings have led to the postulation that the procyclic form lacks the mitosis-cytokinesis checkpoint. However, in light of the differences in positioning the basal bodies between the two life cycle forms and the dominant role of basal body segregation in controlling trypanosome cell division, such a drastic distinction in the mitosis-cytokinesis coordination may stem from the intrinsic cell structure differences between the two forms (32). Other cell structural differences between the procyclic form and the bloodstream form include the positioning of the new flagellar tip, the morphology of the posterior end of the new-flagellum daughter cell during the preabscission stage of cytokinesis, and the structure of the cytoplasmic bridge that connects the two dividing daughter cells during late stages of cytokinesis (8). However, it remains unclear whether and how these structural differences may influence cytokinesis.

Functional analyses of three cytokinesis initiation regulators, CIF1, CIF2, and CIF3, showed that their subcellular localizations and biological functions are conserved in both the procyclic form and the bloodstream form (Fig. 1, 2, and 3). These findings suggest that the initiation of cytokinesis in the bloodstream form also occurs from the distal tip of the new FAZ, despite the fact that the distal tip of the new flagellum is positioned differently from that in the procyclic form (26, 27). The three CIF proteins are interdependent for localization to the new FAZ tip in the bloodstream form (Fig. 6, 7, and 8), but their interdependency in the procyclic form differs substantially (19, 21). In the procyclic form, CIF1 and CIF2 are interdependent for protein stability but not for subcellular localization (19). Additionally, CIF1 is required for maintaining CIF3 protein stability, but CIF3 is required for CIF1 localization to the new FAZ tip (21). Finally, CIF2 is required for maintaining CIF3 protein stability, but CIF3 is not required for CIF2 localization and stability (21). The distinctions in the interdependency among the three cytokinesis initiation regulators between the two life cycle forms suggest that they are regulated by different mechanisms at different life cycle stages. Given that these proteins all localize to the new FAZ tip in both the procyclic form and the bloodstream form and there appears to be no detectable difference in the new FAZ tip structure, such distinctive regulations in protein stability and subcellular localization are, however, unlikely to be related to the morphological differences between the two forms in the T. brucei life cycle.

Previous results showed that knockdown of CIF1 or CIF2 in the procyclic form triggered an alternative cytokinesis pathway, leading to cleavage furrow ingression from the nascent posterior end of the old-flagellum daughter cell (18, 19, 33). The posterior cleavage furrow appears to ingress along the same division fold as the canonical anterior cleavage furrow, albeit in an opposite direction (18). However, such an intriguing phenomenon was not observed in the CIF1- and CIF2-deficient bloodstream trypanosomes (Fig. 1 and 2). The underlying mechanism for this distinction is unclear. Prior to cytokinesis initiation, bloodstream-form trypanosomes also form a division fold between the two flagella, similar to the procyclic form, but the nascent posterior tip of the old-flagellum daughter cell in the bloodstream form is located closer to the nascent posterior of the new-flagellum daughter cell than in the procyclic form (8). It is unclear whether the positioning of the nascent posterior influences the ingression of the cleavage furrow from the posterior cell end. An alternative explanation for the formation of the posterior cleavage furrow in CIF1- and CIF2-deficient procyclic trypanosomes is that remodeling of subpellicular microtubules and plasma membranes at the nascent posterior of the old-flagellum daughter caused the separation of the nascent posterior from the new-flagellum daughter cell body. However, the nascent posterior in the procyclic form and the bloodstream form appears to undergo a similar pattern of microtubule remodeling and morphogenesis (8). Thus, if microtubule remodeling accounts for the formation of the posterior cleavage furrow, both the procyclic form and the bloodstream form should be able to form the posterior cleavage furrow in CIF1 RNAi and CIF2 RNAi cells. However, the fact that only the CIF1- and CIF2-deficient procyclic cells initiated cytokinesis from the posterior end suggests that the remodeling of the nascent posterior is unlikely to contribute to the alternative cleavage furrow ingression.

The recent identification of two trypanosome-specific proteins, KLIF and FRW1, that mark the cell division fold and the cleavage furrow in the procyclic form (20, 22), suggests potentially novel mechanisms in cleavage furrow ingression in T. brucei. KLIF contains a kinesin motor domain at the N terminus and two tropomyosin-like motifs at the C terminus, and FRW1 contains five coiled-coil motifs (20). Knockdown of KLIF in the procyclic form only resulted in a moderate growth defect and caused a defect in the completion of cytokinesis (22). RNAi-mediated ablation of KLIF in the bloodstream form also resulted in a moderate growth defect but did not affect any stages of cytokinesis (Fig. 5), despite the fact that the protein is localized to the new FAZ tip prior to cytokinesis initiation and to the cleavage furrow during cytokinesis (Fig. 5A). It appears that KLIF does not play an essential role in cytokinesis in the bloodstream form but is implicated in cytokinesis completion in the procyclic form. The FRW1 protein, on the other hand, is essential for cytokinesis initiation in the bloodstream form but does not play an essential role in the procyclic form (Fig. 4). The distinct functions of KLIF and FRW1 between the procyclic form and the bloodstream form suggest that the cytokinesis regulatory pathway differs substantially between the two life cycle stages of T. brucei.

## MATERIALS AND METHODS

### Trypanosome cell culture and RNAi.

The bloodstream-form single marker (SM) cell line of Trypanosoma brucei (34) was cultured in HMI-9 medium supplemented with 10% fetal bovine serum and 2.5 µg/ml G418 at 37°C with 5% CO_2_. RNAi of CIF1, CIF2, CIF3, KLIF, and FRW1 was carried out using the pZJM vector (35). The plasmids pZJM-CIF1, pZJM-CIF2, and pZJM-CIF3 have been reported previously (18, 19, 21). To construct pZJM-KLIF, a 548-bp DNA fragment (nucleotides 1143 to 1690) of the *KLIF* gene was PCR amplified using the following primers: forward primer, 5′-ATCTAGCCCCTCGAGATTAAACTTGCCCAAACAGA-3′; and reverse primer, 5′-TTCGATATCAAGCTTAGCTCTAAGTCAGCATCAGC-3′. The PCR fragment was cloned into the XhoI/HindIII sites of pZJM. To construct pZJM-FRW1, a 503-bp DNA fragment (nucleotides 3384 to 3886) of the *FRW1* gene was PCR amplified using the following primers: forward primer, 5′-ATCTAGCCCCTCGAGGTCAGCACCAACCAACACAC-3′; and reverse primer, 5′-TTCGATATCAAG CTTGCGCAGGAGTCATAGAAAGG-3′. The individual RNAi plasmid was linearized by NotI digestion and electroporated into the bloodstream-form SM cell line according to our published procedures (36). Successful transfectants were selected with 2.5 µg/ml phleomycin and were further cloned by limiting dilution in a 96-well plate. To induce RNAi, the clonal cell line was incubated with 1.0 µg/ml tetracycline. Cell growth was monitored by daily counting of cells with a hemacytometer. At least three clonal cell lines were analyzed. To minimize the off-target effect of RNAi, the DNA fragment used for RNAi was chosen after the sequence was searched against the T. brucei genome database to confirm the uniqueness for the specific gene.

### *In situ* epitope tagging of proteins.

Epitope tagging of proteins at the endogenous locus was performed using the one-step PCR-based method described previously (37). For C-terminal epitope tagging, one PCR primer contains a 100-bp DNA sequence that overlaps the 3′-coding sequence immediately upstream of the stop codon of the gene of interest and a 25-bp DNA sequence that amplifies the selection drug marker-containing plasmid vector. The other PCR primer contains a 100-bp DNA sequence that overlaps the 3′-untranslated region (3′-UTR) immediately downstream of the stop codon of the gene of interest and a 25-bp DNA sequence that amplifies the plasmid vector. For N-terminal epitope tagging, one PCR primer contains a 100-bp DNA sequence that overlaps the 5′-UTR immediately upstream of the initiation codon and a 25-bp DNA sequence that amplifies the plasmid vector. The other PCR primer contains a 100-bp DNA sequence that overlaps the 5′-coding sequence immediately downstream of the initiation codon and a 25-bp sequence that amplifies the plasmid vector. Detailed primer design can be found in published literature (37).

### Western blotting and quantitation of protein band intensity.

Western blotting was performed as described previously (38). An equal number (10^7^) of trypanosome cells at different time points of RNAi were collected by centrifugation, lysed in SDS sampling buffer, and boiled for 5 min. Cell lysate was loaded onto a 10% SDS-PAGE gel, and proteins were separated by gel electrophoresis, transferred onto a polyvinylidene difluoride (PVDF) membrane, and blotted in blocking buffer (3% nonfat milk in phosphate-buffered saline [PBS]). The membrane was then incubated with the primary antibody (anti-HA monoclonal antibody, 1:1,000 dilution; anti-CIF1 polyclonal antibody, 1:1,000 dilution; anti-protein A polyclonal antibody, 1:1,000 dilution; and anti-T. brucei PSA6 (anti-TbPSA6) polyclonal antibody, 1:1,000 dilution) for 1 h at room temperature, washed three times with PBS, and then incubated with horseradish peroxidase (HRP)-conjugated secondary antibody (anti-mouse IgG, 1:1,000 dilution; and anti-rabbit IgG, 1:1,000 dilution). The membrane was washed three times with PBS and then developed using the FluorChem HD2 system (ProteinSimple, Inc.).

Quantitation of protein band intensity on the Western blots was performed using the ImageJ software, and band intensity was normalized with that of the loading control.

### Purification of recombinant CIF2 protein and antibody production.

A 1,344-bp DNA fragment corresponding to the CIF2 N-terminal coding region (amino acids [aa] 9 to 456) containing the EF-hand motifs was amplified from the genomic DNA using the following primers: forward primer, 5′-GGAGATATACATATGGGTGTGATTAAGGCAATGGCTG-3′; and reverse primer, 5′-GTGGTGGTGCTCGAGCTGATTAGGCAGTGATGCACACAC-3′. The PCR fragment was cloned into the pET26 vector, and the resulting plasmid pET26-CIF2 was transformed into Escherichia coli strain BL21 for expression of a hexahistidine-fused CIF2 truncation protein. Recombinant His-tagged CIF2 truncation protein was induced with isopropyl-β-d-thiogalactopyranoside (IPTG), purified through a nickel column, and used for immunizing rabbit at Cocalico Biologicals, Inc. (Reamstown, PA). The anti-CIF2 antibody was purified from crude antiserum according to published procedures (39).

### Immunofluorescence microscopy.

Cells were adhered to glass coverslips and fixed in cold methanol (−20°C) for 20 min or treated with PEME buffer [100 mM piperazine-*N,N*′-bis(2-ethanesulfonic acid) (PIPES), 2 mM EGTA, 0.1 mM EDTA, 1 mM MgSO_4_] supplemented with 0.1% Nonidet-P40 at room temperature for 5 min to prepare cytoskeleton. Methanol-permeabilized cells or PEME-extracted cytoskeletons were incubated with the blocking buffer (3% bovine serum albumin [BSA] in PBS) for 1 h at room temperature before incubating with the primary antibody. The following antibodies were used: anti-CIF1 polyclonal antibody (1:1,000 dilution) (20), anti-CIF2 polyclonal antibody (1:1,000 dilution), anti-protein A (anti-ProtA) polyclonal antibody (1:400 dilution) (Sigma-Aldrich), anti-CC2D polyclonal antibody (1:1,000 dilution) (7), and fluorescein isothiocyanate (FITC)-conjugated anti-HA monoclonal antibody (1:400 dilution) (Sigma-Aldrich). Cells were then incubated with primary antibodies at room temperature for 1 h and then washed three times with PBS containing 0.1% Triton X-100. Except for the FITC-conjugated anti-HA monoclonal antibody, cells were then incubated with Cy3-conjugated goat anti-rabbit IgG at room temperature for 1 h. The slides were mounted in VectaShield mounting medium (Vector Laboratories) containing 4′,6-diamidino-2-phenylindole (DAPI) and examined using an inverted microscope (model IX71; Olympus) equipped with a cooled charge-coupled-device (CCD) camera (model Orca-ER; Hamamatsu) and a PlanApo N 60× 1.42-numerical aperture (NA) differential interference contrast (DIC) objective. Images were acquired and processed using the Slidebook5 software (Intelligent Imaging Innovations, Inc.).

### Scanning electron microscopy.

Scanning electron microscopy was performed essentially as described previously (18). Trypanosome cells were harvested by centrifugation, washed three times with PBS, and settled onto glass coverslips. Cells were fixed with 2.5% (vol/vol) glutaraldehyde in PBS for 30 min in the dark at room temperature. Cells were dehydrated with a series of alcohol concentrations (30%, 50%, 70%, 90%, and 100%) and dried by critical point drying. Coverslips were then coated with an 8-nm metal film (Pt:Pd, 80:20; Ted Pella, Inc.) using a sputter coater (Cressington sputter coater 208 HR; Ted Pella, Inc.), and cells on the coverslip were examined using Nova NanoSEM 230 (FEI). The parameters used were 5 mm for the scanning work distance and 8 kV for the accelerating high voltage.

### Cell counting and statistical analysis.

For counting of cells from immunofluorescence microscopy, images were randomly taken, and all cells in each image were counted. To determine the cell cycle of T. brucei in control and RNAi cells, cells were stained with DAPI to label the nucleus (N) and the kinetoplast (K), and the cells with different numbers of N and K were tabulated. Data were collected from three independent experiments.

For statistical analysis, we used the Student *t* test provided in the Microsoft Excel software. Differences were considered statistically significant at a *P* value of <0.05. The *n* values for each panel in the figures indicate the numbers of independent experiments performed.
